# Novel Associations Between Interleukin-15 Polymorphisms and Post-training Changes of Body Composition Parameters in Young Nonobese Women

**DOI:** 10.3389/fphys.2019.00876

**Published:** 2019-07-05

**Authors:** Krzysztof Ficek, Paweł Ciȩszczyk, Katarzyna Leźnicka, Mariusz Kaczmarczyk, Agata Leońska-Duniec

**Affiliations:** ^1^Faculty of Physiotherapy, The Jerzy Kukuczka Academy of Physical Education in Katowice, Katowice, Poland; ^2^Faculty of Physical Education, Gdansk University of Physical Education and Sport, Gdańsk, Poland; ^3^Faculty of Tourism and Recreation, Gdansk University of Physical Education and Sport, Gdańsk, Poland; ^4^Department of Clinical and Molecular Biochemistry, Pomeranian Medical University in Szczecin, Szczecin, Poland

**Keywords:** Interleukin-15, sport genetics, gene, polymorphisms, physical activity, body composition

## Abstract

Based on the important role of interleukin-15 (IL-15) in human metabolism and, in consequence, in body composition modulation, we examined whether rs1589241 and rs1057972 polymorphisms, analyzed individually or in combination, would influence the effects of a training program. Accordingly, we studied the allele and genotype distribution in a group of 163 young nonobese Caucasian women measured for selected body mass and composition, as well as biochemical parameters before and after the completion of a 12-week endurance training program. After a week-long familiarization stage, low-high impact aerobics were conducted three times a week for 60 min, at an increasing intensity from about 50 to 80% of HRmax. With reference to rs1057972 genotypes, there were two significant genotype × training interactions, in which (i) fat mass percentage (FM%) significantly decreased among the AA homozygotes (*p* = 0.00002) and AT heterozygotes (*p* = 0.00002), and (ii) fat free mass (FFM) increased only among the AT heterozygotes (*p* = 0.0003), whereas in the AA homozygotes there was only a borderline significance (*p* = 0.065). No genotype × training interactions were found for the second rs1589241 polymorphism. Moreover, the carriers of the[T;A] haplotype (when compared with reference haplotype) displayed significant decrease in FM% (*p* = 0.027) and increase in FFM (*p* = 0.014) in response to the applied training program. Our data highlight novel associations between specific *IL-15* genotype and different post-training changes of FM% and FFM parameters. The results suggest that harboring the rs1057972 A allele and/or the [T;A] haplotype is favorable for achieving specific positive training-induced body composition changes.

## Introduction

Interleukin-15 (IL-15) is in the four-helix bundle cytokine family that was singled out due to its ability to stimulate T and B cell proliferation, contribute to maintaining CD8^+^ T cell memory, and activate natural killer (NK) cells (Carson et al., 1994; Grabstein et al., 1994; Armitage et al., 1995; Lügering et al., 1999; Klebanoff et al., 2004). Despite being most commonly known as an immunomodulatory cytokine, this IL-15 has also been demonstrated that its effects are not limited to the immune system (Horseman and Yu-Lee, 1994; Barra et al., 2010). The expression of IL-15 has been observed in various non-lymphoid tissues, particularly in skeletal muscle (Grabstein et al., 1994), where it influences processes ranging from angiogenesis to skeletal muscle hypertrophy (Quinn and Anderson, 2011).

As a result of IL-15’s high expression in skeletal muscle and indications that suggest other cytokines are released from muscle after physical activity, some authors have theorized that IL-15 may function as a myokine modulating body composition by means of an endocrine mechanism (Carbó et al., 2001; Riechman et al., 2004; Quinn et al., 2005; Quinn and Anderson, 2011). In a study of young participants divided into two groups, those with no training and those who completed a 10-week-training program, Riechman et al. (2004) showed IL-15 level increases in plasma following resistance exercise. However, some studies have not replicated this association (Nielsen et al., 2007). Laboratory animal studies reported IL-15 to induce adipose tissue mass, especially visceral fat mass, without reducing muscle mass. Additionally, when IL-15 was administered, white fat consequently decreased, circulating triacylglycerols (TG), which correlated with lower rates of hepatic lipogenesis rate and very low density lipoprotein (VLDL) triacylglycerol content. In humans, plasma IL-15 level is inversely related to total fat mass, trunk fat mass, and percent fat mass (Carbó et al., 2001; Nielsen et al., 2007). Based on these results, it is plausible to infer IL-15’s involvement in adipose tissue regulation and association with obesity (Ye, 2015).

The *IL-15* gene is located in human chromosome 4q31 (Anderson et al., 1995). In a genome-wide study of large and rare copy-number variations associated with obesity, a deletion of a region was revealed which included *IL-15* and mitochondrial uncoupling protein gene (*UPC1*) (Wang et al., 2010). Since *UPC1*’s role in regulating energy homeostasis has already been characterized (Kozak and Anunciado-Koza, 2008), the possible impact of *IL-15* loss due to this mutation was not given additional consideration. Data from cell culture, animal model, human genetic, and human subject studies all suggest IL-15 may also exert positive effects on total body mass as well as bodily composition (Carbó et al., 2001; Riechman et al., 2004; Pistilli et al., 2008; Barra et al., 2010; Quinn and Anderson, 2011). A number of single nucleotide polymorphisms (SNPs) have been isolated within the human *IL-15* gene and studied for associations with obesity and skeletal muscle response to resistance exercise (Pistilli et al., 2008). Recently, two polymorphisms (rs1589241 and rs1057972) have been clearly linked to body mass index (BMI), muscle strength, and predictors of various metabolic syndromes (Riechman et al., 2004; Pistilli et al., 2008). These SNPs, found in the 5′ and 3′ untranslated regions (UTRs) of the *IL-15* gene, seem to regulate *IL-15* expression (Quinn and Anderson, 2011).

On the basis of the aforementioned roles of IL-15 cytokine, we set out to determine whether rs1589241 and rs1057972 polymorphisms, analyzed in isolation or collectively, would impact the effectiveness of an exercise training program. Accordingly, we examined the allele and genotype distribution in young Caucasian women whose body mass and composition met certain criteria, as well as biochemical parameters before and after the completion of a 12-week aerobic training program to investigate potential interaction between genotype and fitness outcomes.

## Materials and Methods

### Ethics Statement

All the procedures followed in the study were approved by the Ethics Committee of the Regional Medical Chamber in Szczecin (Approval number 09/KB/IV/2011) and were conducted ethically according to the principles of the World Medical Association Declaration of Helsinki and ethical standards in sport and exercise science research. Furthermore, the experimental procedures were conducted in accordance with the set of guiding principles for reporting the results of genetic association studies defined by the Strengthening the Reporting of Genetic Association studies (STREGA) Statement. All participants were given a consent form and a written information sheet concerning the study, providing all pertinent information (purpose, procedures, risks, and benefits of participation). The potential participant had time to read the information sheet and the consent form. After ensuring that the participant had understood the information, every participant gave written informed consent (signed consent form) to genotyping with the understanding that it was anonymous and that the obtained results would be confidential.

### Participants

One hundred sixty three Polish Caucasian women (age 21 ± 1 years, height 168 ± 2 cm, body mass 61 ± 2 kg) were included in the study. None of these individuals had engaged in regular physical activity in the previous 6 months. They had no history of any metabolic or cardiovascular diseases. Participants were non-smokers and refrained from taking any medications or supplements known to affect metabolism. Participants were included in a dietary program and on the basis of an individual dietary plan, were asked to keep a balanced diet. The nutritional appointment included a prescription of an adequate diet for individual energy needs and nutritional status, with a food replacement list, in addition to orientation on a healthy diet. The share of macronutrients in the energy pool of the average food rations was as follows: 45–65% of total calories coming from carbohydrates, 10–20% from protein, and 20–35% from fat (increase the intake of unsaturated fats and decrease the intake of saturated fats). Daily cholesterol intake <300 mg and dietary fiber intake >25 g were recommended. The participants kept a food diary every day. Weekly consultations were held during which the quality and quantity of meals were analyzed and, if necessary, minor adjustments were made.

### Body Composition Measurements

Before and after the completion of a 12-week training period, all participants were measured for selected body mass and body composition variables, which were assessed with the bioimpedance method using a Tanita TBF 300 M electronic scale (Arlington Heights, Illinois, United States). The device was plugged in and calibrated to account for the weight of clothing (0.2 kg). Afterward, data regarding age, body height, and sex of the subject were inserted. The subjects then stood on the scale with their bare feet on the designated markers. The device analyzes body composition based on the differences of the ability to conduct electrical current by body tissues (different resistance) due to different water content. Body mass and body composition measurements taken with the use of the “Tanita” electronic scale are as follows: total body mass (kg), fat free mass (FFM, kg), fat mass (FM, kg), fat mass percentage (FM%, %), BMI (kg/m^2^), total body water (TBW, kg), and basal metabolic rate (BMR, kJ, or kcal) (Leońska-Duniec et al., 2018b).

### Biochemical and Hematological Analyses

Fasting blood samples were obtained in the morning from the antecubital vein. Blood samples from each participant were collected in two tubes. For biochemical analyses, a 4.9 mL S-Monovette tube with ethylenediaminetetraacetic acid (K 3 EDTA; 1.6 mg EDTA/mL blood) and separating gel (SARSTEDT AG & Co., Nümbrecht, Germany) were used. For complete blood count, a 2.6 mL S-Monovette tube with K 3 EDTA (1.6 mg EDTA/mL blood) (SARSTEDT AG & Co.) was used. Blood samples for biochemical analyses were centrifuged 300 × *g* for 15 min at room temperature in order to receive blood plasma. Biochemical and hematological analyses were performed before the start of the aerobic fitness training program and repeated at the 12th week of this training program (after the 36th training unit). The analyses were performed immediately after the blood collection. Blood count, including white blood cells (WBC), red blood cells (RBC), hemoglobin (HGB), hematocrit (HTC), mean corpuscular volume (MCV), mean corpuscular hemoglobin (MCH), mean corpuscular hemoglobin concentration (MCHC), and total platelet level (PLT) were obtained using Sysmex K-4500 Hematology Analyzer (TOA SYSMEX, Kobe, Japan). All biochemical analyses were conducted using Random Access Automatic Biochemical Analyzer for Clinical Chemistry and Turbidimetry A15 (BIOSYSTEMS S.A., Barcelona, Spain). Blood plasma was used to determine lipid profile: TG, total cholesterol (TC), high-density lipoprotein (HDL), and low-density lipoprotein (LDL) concentrations. Plasma TG and TC concentrations were determined using diagnostic colorimetric enzymatic method according to the manufacturer’s protocol (BioMaxima S.A., Lublin, Poland). The manufacturer’s stated intra-assay coefficients of variation (CV) of the method were <2.5% and <1.5% for the TG and TC determinations, respectively. HDL plasma concentration was determined using human anti-ß-lipoprotein antibody and colorimetric enzymatic method according to the manufacturer’s protocol (BioMaxima S.A.). The manufacturer’s declared intra-assay CV of the method was <1.5%. Plasma concentrations of LDL were determined using a direct method according to the manufacturer’s protocol (PZ Cormay S.A., Łomianki, Poland). The manufacturer’s declared intra-assay CV of the method was 4.97%. All analysis procedures were verified with the use of multiparametric control serum (BIOLABO S.A.S, Maizy, France), as well as control serum of normal level (BioNormL) and high level (BioPathL) lipid profiles (BioMaxima S.A.) (Leonska-Duniec et al., 2018a).

### Training Phase

Before the training program, the maximum heart rate (HRmax) of each participant was evaluated on the basis of a continuous graded exercise test on an electronically braked cycle ergometer (Oxycon Pro, Erich JAEGER GmbH, Höchberg, Germany) according to the previously described protocol (Kostrzewa-Nowak et al., 2015). Individual heart rate (HR) of all participants was also monitored with HR monitors to control the intensity during the class. All participants were instructed to maintain a HR or relative value of HRmax within pre-designated ranges. The training stage was preceded by a week-long familiarization stage, when the examined women exercised three times a week for 30 min, at an intensity of about 50% of HRmax. After the week-long familiarization stage, the proper training started. Each training unit consisted of a warm-up routine (10 min), the main aerobic routine (43 min), and cool-down phase (stretching and breathing exercise for 7 min). The main aerobic routine was a combination of two alternating styles – low and high impact. Low impact style is comprised of movements with at least one foot on the floor at all times, whereas high impact styles include running, hopping, and jumping with a variety of flight phases. Music of variable rhythm intensity (tempo) was incorporated into both styles. A 12-week program of low-high impact aerobics was divided as follows: (i) 3 weeks (9 training units), 60 min each, at about 50–60% of HRmax, tempo 135–140 beats per minute (BPM), (ii) 3 weeks (9 training units), 60 min each, at 60–70% of HRmax, tempo 140–152 BPM, (iii) 3 weeks (9 training units), 60 min with the intensity of 65–75% of HRmax, tempo 145–158 BPM, and (iv) 3 weeks (9 training units), 60 min with an intensity of 65–80% of HRmax, tempo 145–160 BPM. All 36 training units were administered and supervised by the same instructor (Leonska-Duniec et al., 2018a).

### Genetic Analyses

Buccal swabs were collected with two Copan FLOQSwabs, (Interpath, Heidelberg West, Australia), using methods that involved a member of the research team rubbing swabs up and down against the inside of each participant’s check twenty times, then in the maxillary and mandibular buccal sulcus (the upper and lower furrows between the gingiva and the inner cheek) for ten seconds per side. DNA was extracted using a GenElute Mammalian Genomic DNA Miniprep Kit (Sigma, Germany) according to the manufacturer’s protocol. All samples were genotyped in duplicate using an allelic discrimination assay on a C1000 Touch Thermal Cycler (Bio-Rad, Germany) instrument with TaqMan^®^ probes. To discriminate *IL-15* rs1589241 and rs1057972 alleles, TaqMan^®^ Pre-Designed SNP Genotyping Assays were used (Applied Biosystems, United States) (assay ID: C__8865652_10 and C__8976918_10, respectively), including primers and fluorescently labeled (FAM and VIC) MGBTM probes to detect alleles.

### Statistical Analyses

Allele frequencies were determined by gene counting. An *χ*^2^ test was used to test the Hardy-Weinberg equilibrium. To test the influence of *IL-15* rs1589241 and rs1057972 polymorphisms on training response, the mixed 2 × 2 ANOVA with one between-subject factor (genotype) and one within-subject factor (time: before training versus after training) was employed. Additionally, a normality Kolmogorov–Smirnov test was used to check for data normality, and a *post hoc* Tukey test was applied when interaction was significant and was used to perform pair-wise comparisons. Haplotype analysis was conducted with R^1^ using haplo.stats package and haplo.glm regression function. Percentage change over training was used as the dependent variable, while the IL-15 haplotypes were used as the independent variables. The level of statistical significance was set at *p* < 0.05.

## Results

The genotype frequencies of both *IL15* polymorphisms, rs1589241 (NM_000585.4:c.−222+7188T > A) and rs1057972 (NM_000585.4:c.^*^431A > T), did not differ from their Hardy–Weinberg equilibrium expectations (*p* = 0.655, respectively).

No genotype × training interactions were found for the rs1589241 polymorphism (Table 1). Training adaptation stratified by genotype of the rs1057972 polymorphism is shown in Table 2. We observed a genotype x training interaction for FM% (all models) and FFM (general and dominant model). FM% decreased significantly in the AA homozygotes (24.3 ± 6.2 vs 22.3 ± 7.0, SMD = −0.77, Tukey’s HSD test *p* = 0.00002) and AT heterozygotes (24.7 ± 4.7 vs 23.2 ± 4.9, SMD = −0.65, Tukey’s HSD test *p* = 0.00002). FFM increased significantly only among the AT heterozygotes (45.5 ± 2.7 vs 46.1 ± 2.8, SMD = 0.44, Tukey’s HSD test *p* = 0.0003), whereas among the AA homozygotes there was only a borderline significance (46.1 ± 3.7 vs 46.7 ± 3.7, SMD = 0.43, Tukey’s HSD test *p* = 0.065).

**TABLE 1:** Training adaptation with respect to rs1589241 polymorphism (ANOVA with repeated measures).

**Parameter**	**AA (*n* = 88)**		**AT (*n* = 62)**		**TT (*n* = 13)**		**Genotype × training interaction**		
	**Pre**	**Post**	**Pre**	**Post**	**Pre**	**Post**	**General**	**Dom^†^**	**Rec^‡^**
body mass (kg)	60.6 ± 8.2	59.9 ± 7.9	61.6 ± 6.8	60.8 ± 6.8	58.0 ± 7.6	57.8 ± 8.3	0.460	0.800	0.216
BMI (kg/m^2^)	21.5 ± 2.5	21.3 ± 2.4	22.0 ± 2.4	21.8 ± 2.3	20.8 ± 2.3	20.7 ± 2.4	0.513	0.534	0.261
BMR (kcal)	1448 ± 83	1444 ± 83	1457 ± 70	1450 ± 70	1424 ± 81	1429 ± 81	0.115	0.908	0.054
FM% (%)	23.5 ± 5.6	22.3 ± 5.7	25.0 ± 5.0	23.7 ± 4.9	22.3 ± 6.2	20.0 ± 8.0	0.302	0.588	0.122
FM (kg)	14.6 ± 5.3	13.7 ± 5.3	15.7 ± 4.6	14.7 ± 4.6	13.4 ± 5.2	12.2 ± 6.2	0.874	0.682	0.665
FFM (kg)	45.9 ± 3.4	46.3 ± 3.5	45.8 ± 3.0	46.1 ± 3.0	44.7 ± 2.9	45.8 ± 2.8	0.153	0.973	0.068
TBW (kg)	33.5 ± 2.7	33.9 ± 2.6	33.7 ± 2.5	33.8 ± 2.2	32.8 ± 2.1	33.6 ± 2.0	0.132	0.284	0.205
TC (mg/dL)	170 ± 25	171 ± 28	170 ± 25	167 ± 27	165 ± 26	161 ± 24	0.275	0.107	0.577
TG (mg/dL)^*^	4.33 ± 0.37	4.37 ± 0.34	4.29 ± 0.33	4.34 ± 0.38	4.22 ± 0.39	4.31 ± 0.37	0.870	0.710	0.634
HDL (mg/dL)	66.0 ± 13.6	62.6 ± 13.4	63.3 ± 12.5	58.7 ± 11.5	66.4 ± 10.2	60.1 ± 13.3	0.627	0.409	0.460
LDL (mg/dL)	87.0 ± 20.8	91.5 ± 24.3	91.7 ± 23.0	91.2 ± 23.2	84.3 ± 24.9	85.0 ± 24.1	0.315	0.131	0.766

^†^Dominant model: TT+AT vs AA; ^‡^Recessive model: TT vs AT+AA; ^*^natural logarithm of TG; BMI, body mass index; BMR, basal metabolic rate; FM%, fat mass percentage; FM, fat mass; FFM, fat free mass; TBW, total body water; TC, total cholesterol; TG, triglycerides; HDL, high-density lipoprotein; and LDL, low-density lipoprotein.

**TABLE 2:** Training adaptation with respect to rs1057972 polymorphism (ANOVA with repeated measures.

**Parameter**	**TT (*n* = 50)**		**AT (*n* = 78)**		**AA (*n* = 35)**		**Genotype × training interaction**		
	**Pre**	**Post**	**Pre**	**Post**	**Pre**	**Post**	**General**	**Dom^†^**	**Rec^‡^**
body mass (kg)	60.0 ± 8.5	59.3 ± 8.4	60.9 ± 6.2	60.1 ± 6.0	61.7 ± 9.2	60.8 ± 9.1	0.791	0.695	0.507
BMI (kg/m^2^)	21.3 ± 2.5	21.1 ± 2.5	21.7 ± 2.2	21.5 ± 2.1	22.0 ± 2.8	21.7 ± 2.7	0.740	0.802	0.438
BMR (kcal)	1442 ± 89	1436 ± 88	1450 ± 61	1446 ± 63	1461 ± 95	1454 ± 93	0.862	0.826	0.694
FM% (%)	22.7 ± 5.8	21.9 ± 5.7	24.7 ± 4.7	23.2 ± 4.9	24.3 ± 6.2	22.3 ± 7.0	0.027	0.019	0.042
FM (kg)	14.0 ± 5.6	13.4 ± 5.5	15.2 ± 4.1	14.3 ± 4.3	15.5 ± 6.0	14.1 ± 6.3	0.087	0.069	0.071
FFM (kg)	45.9 ± 3.5	46.0 ± 3.5	45.5 ± 2.7	46.1 ± 2.8	46.1 ± 3.7	46.7 ± 3.7	0.045	0.013	0.429
TBW (kg)	33.4 ± 3.0	33.7 ± 2.7	33.5 ± 2.2	33.8 ± 2.1	33.8 ± 2.7	34.2 ± 2.7	0.877	0.737	0.631
TC (mg/dL)	172 ± 24	174 ± 24	168 ± 25	165 ± 28	169 ± 25	169 ± 31	0.223	0.134	0.770
TG (mg/dL)^*^	4.41 ± 0.42	4.42 ± 0.36	4.28 ± 0.31	4.32 ± 0.36	4.24 ± 0.34	4.34 ± 0.38	0.473	0.383	0.267
HDL (mg/dL)	67.9 ± 14.8	64.6 ± 13.3	63.6 ± 12.6	58.7 ± 12.8	64.1 ± 10.6	60.6 ± 10.9	0.633	0.506	0.680
LDL (mg/dL)	85.7 ± 18.7	92.0 ± 20.6	89.7 ± 23.6	89.8 ± 24.2	90.2 ± 22.9	91.4 ± 27.3	0.222	0.085	0.710

^†^Dominant model: AA+AT vs TT; ^‡^Recessive model: AA vs AT+TT; ^*^natural logarithm of TG; BMI, body mass index; BMR, basal metabolic rate; FM%, fat mass percentage; FM, fat mass; FFM, fat free mass; TBW, total body water; TC, total cholesterol; TG, triglycerides; HDL, high-density lipoprotein; and LDL, low-density lipoprotein.

Table 3 presents the results of haplotype-based analysis. Four rs1589241-rs1057972 haplotypes were inferred: [A;T], [T;A], [A:A], and [T;T] with frequencies of 51.8, 24.2, 21.2, and 2.8%, respectively. Haplotype [T;A] (recessive model) was associated with a decrease in FM% with response to training (coefficient −1.64, *p* = 0.027) compared with individuals homozygous for the reference^®^ [A;T] haplotype. The same haplotype was associated with an increase in FFM under the recessive model (coefficient 1.02, *p* = 0.014) compared with individuals homozygous for the reference [A;T] haplotype (Table 3).

**TABLE 3:** Training adaptation with respect to rs1589241 – rs1057972 haplotypes.

**Parameter**	**Additive**				**Dominant**				**Recessive**			
	**Intercept**	**[A;A]**	**[T;A]**	**[T;T]**	**Intercept**	**[A;A]**	**[T;A]**	**[T;T]**	**Intercept**	**[A;A]**	**[T;A]**	**[T;T]**
body mass (kg)	1.29	–0.28 (0.201)	0.08 (0.705)	–0.15 (0.754)	1.37	–0.36 (0.185)	0.007 (0.979)	–0.06 (0.934)	1.13	–0.36 (0.509)	0.66 (0.209)	–0.66 (0.555)
BMI (kg/m^2^)	0.66	–0.11 (0.125)	0.05 (0.463)	–0.11 (0.475)	0.69	–0.11 (0.227)	0.05 (0.562)	–0.09 (0.703)	0.60	–0.29 (0.097)	0.22 (0.194)	–0.35 (0.328)
BMR (kcal)	39.1	–1.93 (0.488)	1.69 (0.531)	–2.51 (0.671)	40.8	–2.84 (0.404)	–0.68 (0.836)	–2.81 (0.734)	31.0	–3.55 (0.599)	14.7 (0.022)	−7.92 (0.000)
FM% (%)	0.03	–0.62 (0.049)	–0.59 (0.053)	0.29 (0.662)	−0.14	–0.64 (0.098)	–0.41 (0.273)	0.62 (0.509)	0.07	–0.68 (0.372)	−1.64 (0.027)	0.64 (0.687)
FM (kg)	−0.11	–0.45 (0.052)	–0.28 (0.204)	0.10 (0.830)	−0.17	–0.46 (0.098)	–0.25 (0.364)	0.28 (0.685)	−0.25	–0.67 (0.237)	–0.46 (0.294)	0.22 (0.850)
FFM (kg)	3.23	0.27 (0.130)	0.26 (0.126)	–0.40 (0.282)	3.43	0.31 (0.147)	0.12 (0.550)	–0.55 (0.304)	2.90	0.11 (0.793)	1.02 (0.014)	–1.09 (0.218)
TBW (kg)	7.66	0.15 (0.420)	0.02 (0.889)	–0.36 (0.436)	7.71	0.20 (0.408)	–0.12 (0.600)	–0.53 (0.522)	7.39	0.04 (0.937)	0.58 (0.176)	–0.67 (0.471)
TC (mg/dL)	41.2	0.13 (0.964)	–4.42 (0.106)	–1.09 (0.859)	41.0	0.08 (0.982)	–5.19 (0.119)	–1.20 (0.859)	39.3	3.69 (0.593)	–6.47 (0.326)	–0.33 (0.982)
TG (mg/dL)	3.29	0.01 (0.757)	–0.05 (0.283)	0.13 (0.228)	3.27	0.03 (0.594)	–0.05 (0.361)	0.22 (0.169)	3.30	0.02 (0.843)	–0.12 (0.262)	0.22 (0.356)
HDL (mg/dL)	21.3	–1.01 (0.462)	–1.54 (0.250)	–4.65 (0.122)	22.4	–1.98 (0.238)	–2.59 (0.113)	–6.41 (0.137)	19.1	2.47 (0.466)	1.41 (0.662)	–8.40 (0.232)
LDL (mg/dL)	31.9	–0.44 (0.866)	–3.55 (0.164)	0.99 (0.862)	31.4	–0.07 (0.981)	–3.68 (0.237)	1.70 (0.838)	31.2	0.27 (0.966)	–6.77 (0.269)	3.64 (0.785)

*BMI, body mass index; BMR, basal metabolic rate; FM%, fat mass percentage; FM, fat mass; FFM, fat free mass; TBW, total body water; TC, total cholesterol; TG, triglycerides; HDL, high-density lipoprotein; and LDL, low-density lipoprotein.*

## Discussion

In an effort to ascertain whether common *IL-15* polymorphisms have an impact on selected mass and body composition parameters, as well as TC, TG, HDL, and LDL levels, we analyzed the distribution of genotypes, alleles, and haplotypes. The describedrs1589241 and rs1057972 polymorphisms were examined in a group of young nonobese women who took part in a 12-week aerobic training program. The results support the claim that *IL-15* plays an important role in body composition modulation.

The results of our statistical analyses indicated that the presence of a specific *IL-15* genotype might correlate with differing outcomes in response to training. Among rs1057972 genotypes, two significant genotype × training interactions were observed in which (i) FM% significantly decreased in the AA homozygotes and AT heterozygotes, and (ii) FFM increased only in the AT heterozygotes, whereas in the AA homozygotes there was only borderline significance. The results suggest that when specific changes in body composition in response to training are considered, carrying the AA or AT genotype might impart an advantage. In contrast, the TT genotype seems to be an unfavorable factor with regards to the effectiveness of training in yielding body composition changes. Provided muscle-derived IL-15 is capable of lipolysis production, it could be the case that the presence of the A allele modifies plasma IL-15 levels, resulting in lipolysis reduction and, in turn, a FM% decrease and FFM increase. Interactions of genotype × training were not found for the second rs1589241 polymorphism.

One novel finding emerged when a complex haplotype analysis was performed using the data from this study, specifically that harboring the [T;A] haplotype (when compared with reference haplotype) displayed significant FM% reduction and elevated FFM in response to the applied training program. The next significant insight from the current study suggests that carriers of this specific [T;A] haplotype have more favorable outcomes with regards to obtaining improved body composition. These associations are intriguing with respect to muscle-derived IL-15’s potential role in modulating lipolysis and glycogenolysis. However, the mediating role of the molecular mechanisms of this cytokine and their effect on adipose tissue have yet to be fully explained. Almendro et al. (2009) suggested that IL-15 upregulates calcineurin mRNA expression, thus hindering adipose cell differentiation (Quinn and Anderson, 2011).

Numerous cytokines are activated within skeletal muscle tissue after physical exertion, affecting the plasma myokine levels. Nieman et al. (2003) showed that in skeletal muscle, IL-15 was the mostly highly expressed cytokine measured at the level of mRNA (Quinn and Anderson, 2011). As both IL-15 and exercise yield beneficial outcomes in terms of improving body mass and composition parameters, it was hypothesized that previous training or state of physical fitness might mediate the supposed release of IL-15 within skeletal muscle tissue in response to exercise (Pedersen, 2009). In a study of 153 participants who completed a 10-week resistance exercise training program, Riechman et al. (2004) showed increased levels of IL-15 concentration in plasma immediately after resistance exercise, speculating that the cytokine was released through physical activity by means of micro tears in muscle fibers (Quinn and Anderson, 2011). Contrary to these findings, a similar study using healthy, young male volunteers who performed high intensity resistance exercise involving only the quadriceps muscle, no elevation of muscle or circulating IL-15 levels were revealed at intervals from 6 to 48 h afterward; however, heightened IL-15 mRNA expression was seen in the quadriceps muscle 24 h after exercise (Nielsen et al., 2007; Banitalebi et al., 2019). There is a lack of consistency in the findings as to whether a training program influences IL-15 expression in skeletal muscle and/or increasing IL-15 levels in blood, however, the inverse relationship between plasma IL-15 concentration and body mass and composition parameters is well confirmed. Barra et al. described obese participants with lower plasma IL-15 levels than lean people (Barra et al., 2010; Pedersen, 2013). This cytokine was also considered to be a local factor implicated in muscle hypertrophy and inhibiting lipogenesis. Nielsen et al. demonstrated that a high IL-15 level correlates with lower BMI, total fat mass, trunk fat mass, and limb fat mass (Nielsen et al., 2007). These findings were supported by Tamura et al. who suggested that IL-15 might be involved in mediating systemic and local benefits as a result of endurance exercise, specifically insulin sensitizing and limiting adipogenesis as well as anabolic processes in skeletal muscle (Tamura et al., 2011). These studies seem to indirectly support the described association between *IL-15* polymorphisms and FFM changes induced by endurance training. However, the mechanism of these reactions is still unknown and further investigation is needed.

Two polymorphisms (rs1589241 and rs1057972) suggested to modulate IL-15 expression, were associated with TC, LDL, glucose levels, BMI, and muscle strength (Riechman et al., 2004; Pistilli et al., 2008). Our findings did not reveal any correlation between the polymorphism variants and biochemical profile. One possible explanation relies on population-specific characteristics, which include generally high PA levels and comparatively small body mass in the studied population. Another possible factor includes a lack of sufficient statistical power given the limited number of participants and low occurrence of the minor alleles in the studied cohort. Finally, the SNPs selected for study may also play a role. However, our results confirmed that the rs1057972 A allele and the [T;A] haplotype are associated with body composition parameters, such as FM% and FFM, however, it is inconsistent with other authors’ findings. Pistilli et al. (2008) showed that the presence of the rs1589241 T allele correlated with higher BMI levels and homeostatic model assessment (HOMA) in male participants, as well as greater TC and LDL levels in women. Additionally, the presence of the rs1057972 A allele correlated with higher fasting glucose values and greater BMI in men (Pistilli et al., 2008). There could be numerous reasons for discrepancies between these studies: (i) heterogeneity between study populations, (ii) varying diets, (iii) discrepancy in training program and methods of physical activity measurement, and (iv), relatively small sizes of study groups which might lack the statistical power necessary to conduct meaningful analysis and interpretation (Leonska-Duniec et al., 2016). What is more, Pistilli et al. (2008) showed that numerous *IL-15* genotype × training interactions were gender-specific. Unfortunately, our study was focused only on women, because men did not report to the experiment, so we could not observe these dependences.

In summary, our findings indicate previously unidentified associations between specific *IL-15* genotypes and different post-training changes of FM% and FFM parameters. The results suggest that harboring the rs1057972 A allele and/or the [T;A] haplotype is favorable for achieving the desired body composition changes as a result of training. These associations are intriguing with respect to muscle-derived IL-15’s potential to affect lipolysis and glycogenolysis. One of the primary objectives of exercise genomics is to identify molecular markers which independently, or in conjunction with other biological factors, could be used to project the potential success of training programs. Broadening our knowledge of the genetic background of physiological processes should greatly enhance the ability of those in the field of exercise science to individualize exercise so that it is more efficient and safer, as well as improve recovery support, traumatology, medical care, diet, supplementation, and many other areas (Leonska-Duniec et al., 2016).

## Ethics Statement

The study was approved by the Ethics Committee of the Regional Medical Chamber in Szczecin (Approval Number 09/KB/IV/2011).

## Author Contributions

AL-D and PC conceived and designed the experiments. AL-D, KF, and KL performed the experiments. MK performed the statistical analysis. AL-D and PC analyzed and interpreted the data. KF and AL-D wrote or provided the critical revision on the manuscript.

## Conflict of Interest Statement

The authors declare that the research was conducted in the absence of any commercial or financial relationships that could be construed as a potential conflict of interest. The reviewer MN and handling Editor declared their shared affiliation at the time of the review.

## Footnotes

1 https://cran.r-project.org, version 3.1.0
